# High-density DArTSeq SNP markers revealed wide genetic diversity and structured population in common bean (*Phaseolus vulgaris* L.) germplasm in Ethiopia

**DOI:** 10.1007/s11033-023-08498-y

**Published:** 2023-06-30

**Authors:** Yonas Moges Gelaw, John S. Y. Eleblu, Kwadwo Ofori, Berhanu Amsalu Fenta, Clare Mukankusi, Ermias Assefa Emam, Samuel Offei

**Affiliations:** 1 Haramaya University, Dire Dawa, Ethiopia; 2 West Africa Centre for Crop Improvement, University of Ghana, Legon, Accra, Ghana; 3 Melkassa Agricultural Research Center, Ethiopian Institute of Agricultural Research (EIAR), Adama, Ethiopia; 4 International Centre for Tropical Agriculture (CIAT), Kawanda, Kampala, Uganda; 5 Bio and Emerging Technology Institute, Addis Ababa, Ethiopia

**Keywords:** DArTSeq, Genetic diversity, Heterozygosity, PIC, SNP markers

## Abstract

**Introduction:**

Common bean is one of the widely consumed food security crop in Africa, Asia, and South America. Understanding genetic diversity and population structure is crucial for designing breeding strategies.

**Materials:**

Two hundred and eighty-nine germplasm were recently collected from different regions of Ethiopia and introduced from CIAT to estimate genetic diversity and population structure using 11,480 DArTSeq SNP markers.

**Results:**

The overall mean genetic diversity and polymorphic information content (PIC) were 0.38 and 0.30, respectively, suggested the presence of adequate genetic diversity among the genotypes. Among the geographical regions, landraces collected from Oromia showed the highest diversity (0.39) and PIC (0.30). The highest genetic distance was observed between genotypes collected from SNNPR and CIAT (0.49). In addition, genotypes from CIAT were genetically more related to improved varieties than the landraces which could be due to sharing of parents in the improvement process. The analysis of molecular variance revealed that the largest proportion of variation was due to within the population both in geographical region (63.67%) and breeding status (61.3%) based classification. Model-based structure analysis delineated the 289 common bean genotypes into six hypothetical ancestoral populations.

**Conclusions:**

The genotypes were not clustered based on geographical regions and they were not the main drivers for the differentiation. This indicated that selection of the parental lines should be based on systematic assessment of the diversity rather than geographical distance. This article provides new insights into the genetic diversity and population structure of common bean for association studies, designing effective collection and conservation for efficient utilization for the improvement of the crop.

**Supplementary Information:**

The online version contains supplementary material available at 10.1007/s11033-023-08498-y.

## Introduction

Common bean (*Phaseolus vulgaris* L.) is a diploid (2n = 2x = 20) annual leguminous self-pollinated crop with estimated genome size ranged from 588 to 637 Mbp [1, 2]. It is an important component of agrobiodiversity to provide food, feed, cash income and improve soil fertility through its mutual association with rhizobium bacteria, thereby reducing the costs of chemical fertilizer and its negative impacts on the environment [3]. Thus, contribute to improved livelihood of smallholder farmers throughout the tropics [4, 5].

Africa is considered as the secondary center of diversity of common bean [6]. Common bean is one of the food security and commercial crops in Ethiopia widely cultivated in all regions. It largely contributes to food and nutrition security and income generation for smallholder farmers [7]. It is one of the main components of nutrition sensitive agriculture which works to significantly reduce malnutrition from Ethiopia in 2030 [8]. In Ethiopia, it is grown by over 3.95 million households and is one of their main source of income. Between 2004 and 2017, income from bean exports increased by over seven-fold from USD 18 to 130 million. It is produced in an area of 355,550 ha and a production of 619,094.4 tonnes with a productivity of 1.74 ton ha^−1^. The country stands 11th in area and 4th in production among the 36 countries that produce common bean in sub-Saharan Africa [9].

The productivity of common bean is low due to biotic and abiotic factors. The improved varieties are mainly introductions from other countries and do not adapted well to the diverse soil and climatic conditions of the country. On the other hand, landraces are well adapted to the local condition and sources for useful trait. Common bean cultivars found in the hands of the farmers are very diverse and the crop plays a diversified role within the Ethiopian farming system and food culture [10]. It has a wider importance in the lowlands of Ethiopia because of its better adaptability to the persistent warmer climates and prevalent disease pressures. Landraces are an important component of agrobiodiversity; however, most of them are at risk of extinction because of climate change and replacement with improved varieties. The genetic diversity present among the common bean genotypes conserved in the gene bank and landraces cultivated by the farmers can be a source of useful traits to be exploited by breeding programs. Collection and characterization of common bean germplasm from the different regions of Ethiopia should be considered as a high priority. Information on the genetic diversity and population structure of common bean landraces helps to preserve biodiversity and better management of the available genepool. It also helps, for better exploitation of genetic resources in breeding programs, to develop common bean varieties that increase productivity.

Phenotypic characterization and analysis of pedigree information are widely used in the improvement of common bean but are less efficient in the selection of elite materials due to environment and genotype x environment interaction. To overcome the challenge and accelerate the improvement efficiency, molecular markers could be used as a complement to accelerate the common bean breeding.

To use molecular markers in the diversity study, there needs to identify markers that are cheap, easy to work with, and have high reproducibility. Thus, various molecular markers have been used to study common bean genetic diversity and population structure: RFLP [11]; AFLP [12, 13]; RAPD [14, 15]; SSR [16-18]; ISSR [19]. However, these markers have limitations associated with poor genome coverage, low marker density, and cost ineffectiveness [20]. Recently, SNP markers are widely used in common bean diversity study, population structure, and genome-wide association studies (GWAS) [21–24]. In Ethiopia, limited studies were conducted to characterize common bean germplasm using small number of germplasm and molecular markers such as ISSR [23, 25]; SSR [26]; SNP [24].

DArTSeq SNPs have been used in many cereal crops: sorghum [27], maize [28, 29], durum wheat [30], and legume crops: cowpea [31] and chickpea [32]. However, such studies are limited in common bean. Therefore, the objective of this study was to investigate the extent and pattern of genetic diversity and population structure of common bean genotypes using high-density DArTSeq SNP markers.

## Materials and methods

### Plant material

The present study consisted of 300 common bean genotypes including recent collections by Lowland Pulse Research Program of Melkassa Agricultural Research Center (MARC) from major common bean-growing areas of Ethiopia, Ethiopian Biodiversity Institute (EBI), improved varieties and genotypes from International Centre for Tropical Agriculture (CIAT). Information on the geographical description such as name of the locations and altitude was collected during the collection process. From the 164 common bean landraces, 85 were collected from Amhara, 38 from Oromia, 29 from Southern Nations and Nationalities and Peoples’ Region (SNNPR), 10 from Benshangul Gumuz, and two from Somali region. The collection site of the genotypes is depicted in Table S1. In addition, 62 improved and pipeline varieties and 74 introductions from CIAT were included in the study. The improved and pipeline varieties were sourced from Haramaya University and Agricultural Research centers such as Melkassa, Hawassa, and Sirinka, while introductions were received from CIAT.

**Fig. 1:**
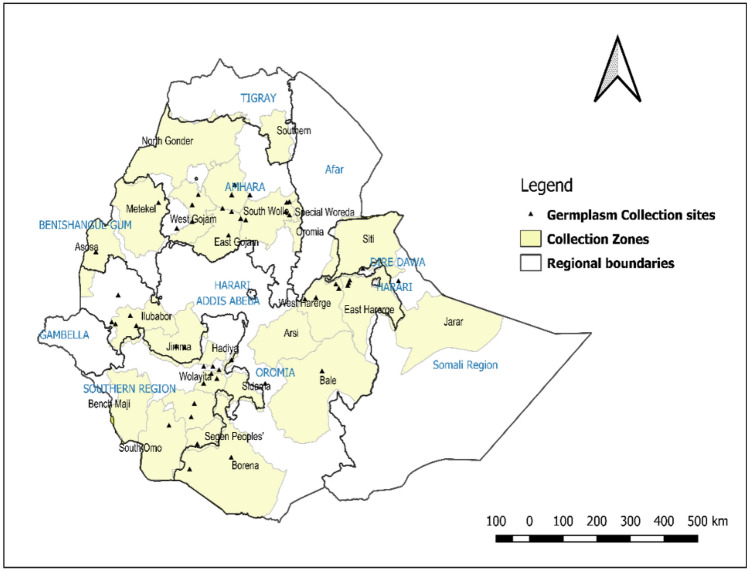
Collection sites of common bean landraces in Ethiopia.

All the common bean materials used in this study, hereafter called genotypes, were regarded as a population and each grouping based on geographic regions and breeding status was regarded as subpopulation. The detailed description of the collection site is presented in Fig. 1.

### DNA extraction and sequencing

Four representative seeds of each common bean genotypes were grown in a germination chamber. DNA extraction was done from a pooled sample of two-week-old seedling’s leaf samples. The leaf samples were stored at − 80 ^O^c and genomic DNA was extracted from frozen leaf tissue using a modified CTAB protocol. The DNA quantity was checked using NanoDrop Spectrophotometer 2000c (ND-2000 v3.5, NanoDrop Technologies Inc). The DNA concentration was quantified with a Qubit fluorometer (Life Technologies, Carlsbad, CA, USA) followed by running 0.8% agarose gel electrophoresis. The DNA concentration was adjusted to 50–100 ηg µl^−1^ and sent for sequencing using GBS technology as described by [33] using DArTSeq™ technology platform in Biosciences eastern and central Africa** (**BecA**)-**ILRI Hub, Nairobi, Kenya.

High-throughput DArTseq technology was applied to genotype the common bean genotypes. Complexity reduction was done using Pstl to enrich genomic representations with single-copy sequences. Polymerase chain reaction (PCR) adapters were ligated to the Pstl fragment ends followed by PCR amplification of adapter-ligated fragments. Genotype by sequencing was conducted using a combination of DArT complexity reduction methods and next-generation sequencing (NGS) following [34]. Next-generation sequencing was carried out using Illuminia Hiseq2500. DArTSeq markers scoring was achieved using DArTsoft14 software plugin in KDCompute application (http://www.kddart.org/kdcompute.html). Two types of DArTSeq markers were scored, silicoDArT and SNP markers. Both markers were aligned to the Common bean_v9 reference genome of common bean to identify the chromosome positions.

### Data filtering process and DArTSeq SNP calling

Initially, we received 24,191 single-row SNP markers from DArT Pty Ltd., which were polymorphic across common bean genotypes and distributed across the 11 chromosomes. Markers with unknown position were first removed from the analysis. The parameters such as call rate and MAF were determined in TASSEL platform v5.2.65. Accordingly, SNPs with ≥ 80% call rate and MAF of ≥ 0.05 were retained for further analysis. In addition, genotypes with missing values of ≥ 30% were removed from the analysis. Accordingly, 289 genotypes and 11,480 polymorphic SNPs were used for downstream analysis (Table S2).

### Population structure and genetic diversity analysis

Population stratification into genetically similar groups was performed using STRUCTURE software v2.3.4 [35] based on Bayesian clustering model. The burn-in period of 50,000 and a Monte Carlo Markov Chain (MCMC) iteration of 100,000 were used by considering an admixture model with allele frequencies uncorrelated. The number of clusters was set using *K* values between 2 and 10 with 10 iterations. The optimal *K* value was detected by ΔK [36] using a web-based structure harvester software http://taylor0.biology.ucla.edu/structureHarvester/). Clusters were aligned from the independent runs using the in-file generated by structure harvester using CLUMPP server [37]. DISTRUCT v1.1. was used to generate bar plots from average runs for the most probable K value [38].

### Genetic relationship and summary statistics

Genetic distance and kinship matrix of 289 pairwise common bean genotypes based on 11,480 SNPs markers were calculated using Identity By State (IBS) method implemented in TASSEL v5.2.64 [39]. Diversity-relevant metrics such as expected heterozygosity (He), overall genetic diversity (GD), polymorphic information content (PIC), major allele frequency, and inbreeding coefficient (Fis) were computed using PowerMarker v3.25 software [40]. Arlequin v3.5.2.2. Software [41] was used to estimate the genetic variation between and within geographical regions and breeding status through the analysis of molecular variance.

For the estimation of genetic differentiation and relationships, distance between the genotypes and between pre-grouped populations were measured based on shared allele distances using the KD compute plugin system https://kdcompute.seqart.net/kdcompute/login. Clustering of the genotypes and PCA for the genetic relationships was computed using the allele-sharing matrix using KDCompute plugin system. Principal coordinate analysis was computed using GenAlEx software V6.5 [42].

## Results

### SNP marker variation and distribution

Whole-genome DArTSeq sequencing of common bean genotypes yielded 24,191 silicoDArT markers which were reproducible and aligned to the common bean reference genome v9 [43]. Among them, only the positions of 22,067 (91.2%) markers were provided. Scaffolds (170) and other markers (1954) were not located into any of the chromosomes.

In addition, genotypes were filtered for call rate and seven genotypes with ≤ 30% call rate were removed and only 289 genotypes were used for the analysis. Based on these criteria, the remaining 11,480 polymorphic and high-quality SNPs distributed across the 11 common bean chromosomes were used for further analysis (Table S2).

**Fig. 2:**
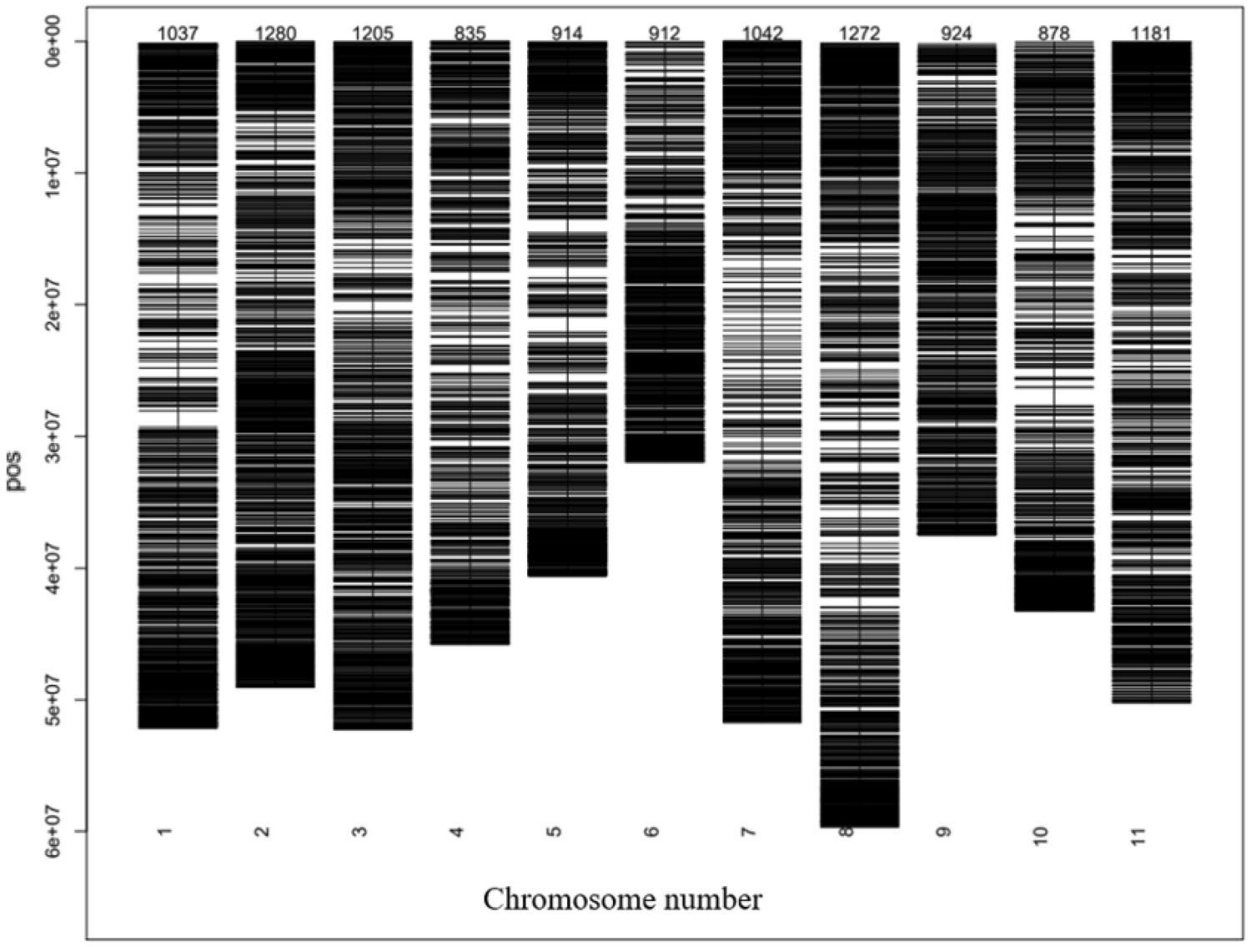
Distribution of SNP markers across the common bean chromosomes.

The highest and lowest marker densities were observed on chr_2 (1280 SNPs) and on chr_4 (835 SNPs), respectively, with an average of 1044 markers per chromosome (Fig. 2). The average call rate and reproducibility of these markers were 0.94 and 1.00, respectively (Table 1). The average call rate, one ratio, heterozygosity, and PIC were consistent across the chromosomes.

**Table 1:** Quality estimates of DArTSeq-derived SNP markers across the common bean chromosomes

Chromosome	Call rate	One ratio	Heterozygosity	Polymorphic information content	Average reproducibility
Chr_1	0.94	0.52	0.04	0.28	1.00
Chr_2	0.95	0.52	0.04	0.29	1.00
Chr_3	0.94	0.52	0.04	0.30	1.00
Chr_4	0.94	0.52	0.04	0.30	0.99
Chr_5	0.94	0.52	0.04	0.30	0.99
Chr_6	0.94	0.52	0.04	0.30	0.99
Chr_7	0.94	0.52	0.04	0.30	1.00
Chr_8	0.94	0.52	0.04	0.30	0.99
Chr_9	0.94	0.52	0.04	0.30	1.00
Chr_10	0.93	0.52	0.04	0.30	0.99
Chr_11	0.93	0.52	0.05	0.32	0.99
Mean	0.94	0.52	0.05	0.30	1.00

In this study, more transitions (58.23%) than transversions type SNPs (41.77%) were observed with a transition to transversion (Ts/Tv) ratio of 1.39:1(6685/ 4795). It was also found that the C/T and A/G transitions were more than the T/C and A/G transitions. On the other hand, more transversions were observed in A/T, T/A, G/T, and C/T than G/C, T/G, A/C, and C/G.

**Fig. 3:**
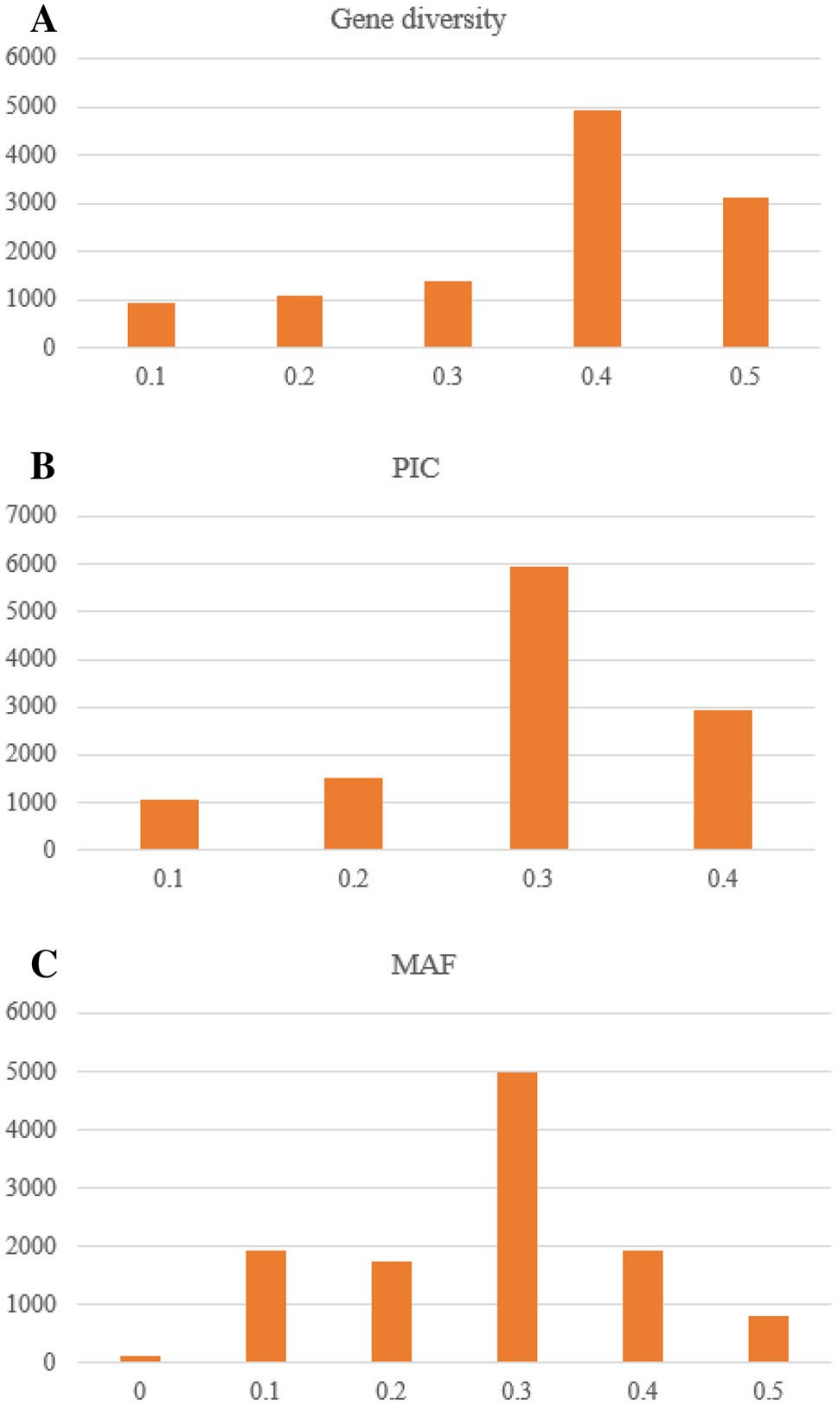
Frequency distribution of genetic diversity, polymorphic information content (PIC), and Minor allele frequency (MAF) of 11,480 polymorphic SNPs for the 289 common bean germplasm.

The overall mean genetic diversity of the genotypes was 0.38 which ranged from 0.07 to 0.50 (Table 2). Polymorphic information content varied from 0.06 to 0.38 with an average of 0.30. Minor allele frequency (MAF) varied from 0.03 to 0.50 with a mean of 0.28. The mean inbreeding coefficient was 0.85. Single-nucleotide polymorphism markers exhibited a wide range of genetic diversity, PIC, and MAF across the common bean chromosomes (Fig. 3).

**Table 2:** Diversity estimates of 289 common bean genotypes using 11,480 DArTSeq SNP markers

Parameter	Minimum	Maximum	Mean
Genetic diversity	0.07	0.50	0.38
Polymorphic information content	0.06	0.38	0.30
Minor allele frequency	0.03	0.50	0.28
Heterozygosity	0.00	0.80	0.05
Reproducibility	0.9	1	1.00
Inbreeding coefficient	−0.65	1.00	0.85

### Genetic divergence based on geographic origin and breeding status of the genotypes

#### Genetic diversity based on geographic origin

Among the geographical regions, the highest genetic diversity (0.39) was found in the landraces collected from Oromia region, followed by genotypes from CIAT (0.32), while the lowest diversity was obtained in the Benshangul Gumuz collections (0.11) (Table 3). The maximum heterozygosity (0.08) was found from the CIAT collections. Polymorphic information content across the geographical regions ranged from 0.11 (Benshangul Gumuz) to 0.30 (Oromia). The value of inbreeding coefficient (FIS) ranged from 0.72 for the landraces collected from Benshangul Gumuz to 0.88 for landraces collected from Oromia and improved varieties.

**Table 3:** Estimates of major allele frequency, gene diversity, heterozygosity, PIC, and inbreeding coefficient of 287 common bean germplasm classified based on geographic location and breeding status

Population	No. of genotype	genetic diversity	Heterozygosity	PIC
Amhara	81	0.27	0.04	0.23
Benshangul Gumuz	10	0.13	0.04	0.11
CIAT	74	0.32	0.08	0.26
Improved varieties	59	0.32	0.04	0.26
Oromia	38	0.39	0.05	0.30
SNNPR	25	0.14	0.04	0.12
Landrace	154	0.27	0.04	0.23
Improved varieties	133	0.37	0.06	0.29
Mean		0.38	0.05	0.30

*CIAT*: International Centre for Tropical Agriculture, *PIC*: Polymorphic Information Content, *SNNPR*: Southern Nations Nationalities and People’s Region

#### Genetic divergence between landraces and improved varieties

Genetic diversity of the landraces and improved varieties was 0.27 and 0.37, respectively, with a mean of 0.38. (Table 3). Heterozygosity was low both for the landraces (0.04) and for the improved varieties (0.06). Improved varieties showed higher (0.29) PIC as compared with the landraces (0.23).

### Genetic relationship among genotypes

**Fig. 4:**
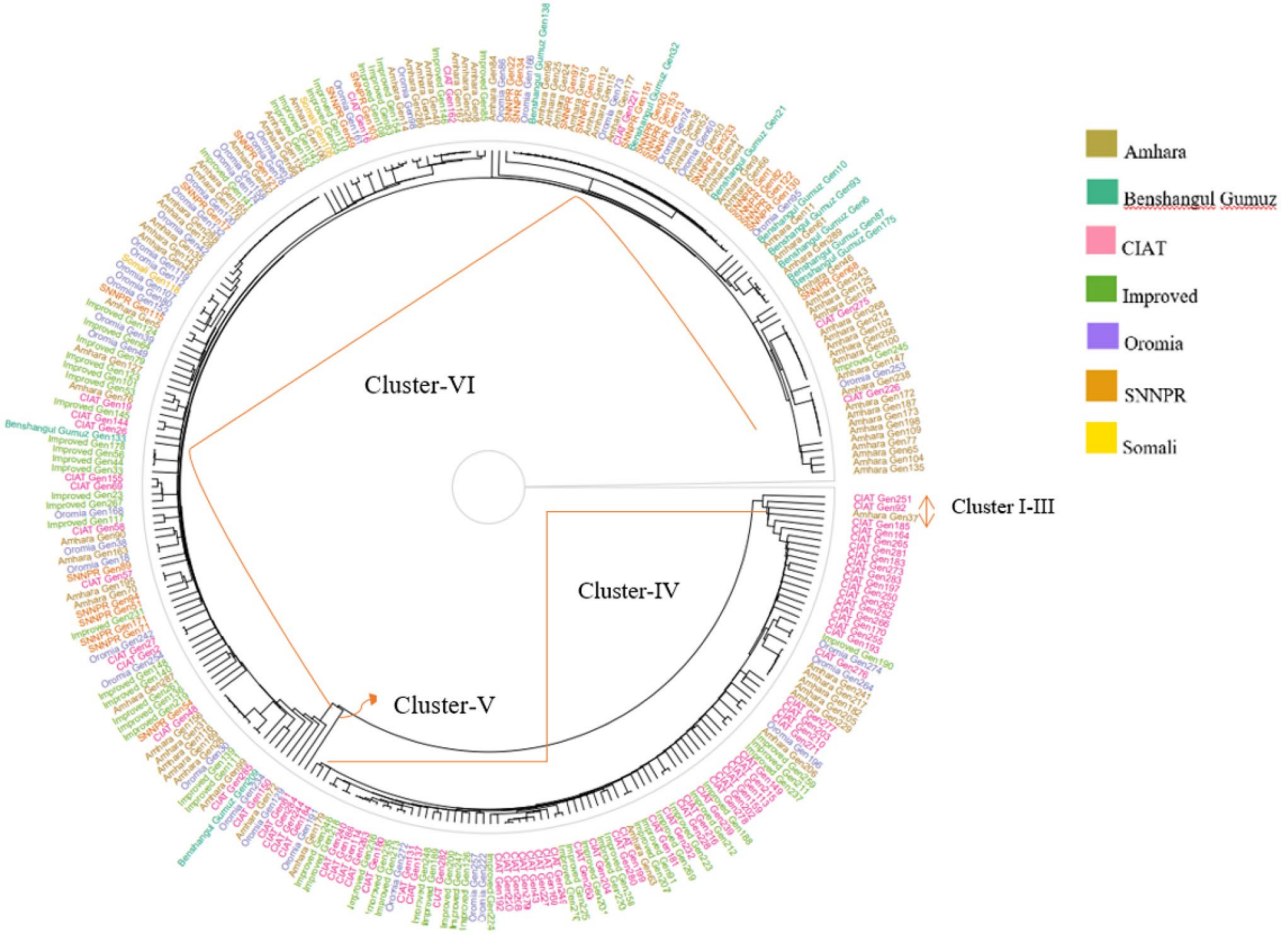
Clustering of 289 common bean germplasm using Euclidean distance. The regions of collection and genotype are represented by different colors.

Pre-grouping of the genotypes into the different regions of collection and improved varieties revealed that the highest genetic distance was observed between SNNPR and CIAT (0.49), while genotypes from Oromia and Amhara showed close similarity (0.03) (Table 4). In general, genotypes from CIAT were closer to the improved varieties compared with landraces collected from Ethiopia. Grouping of genotypes revealed six distinct clusters. The result also showed that the genotypes were not clustered based on their geographical location, which could be due to the exchange of genetic materials across the geographic regions (Fig. 4).

**Table 4:** Pairwise Ne’s genetic distance showing genetic differentiation among the different regions of common bean collection sites

Geographic area	Amhara	Benshangul Gumuz	CIAT	Improved	Oromia	SNNPR
Amhara	0					
Benshangul Gumuz	0.1	0				
CIAT	0.38	0.47	0			
Improved	0.16	0.24	0.10	0		
Oromia	0.04	0.13	0.30	0.10	0	
SNNPR	0.08	0.07	0.49	0.27	0.13	0

### Analysis of molecular variance

In the present study, the analysis of molecular variance of the common bean genotypes based on pre-defined geographic regions and breeding status is presented in Table 5. The result revealed that the largest proportion of variation was due to within populations based on both geographical region (63.8%) and breeding status (61.3%) classifications. Similarly, variation among populations accounted for 23.58% and 27.06% was due to geographical regions and breeding status, respectively. Fixation index, a measure of population differentiation, revealed high fixation index of 0.88 and 0.87 for within individual’s variations for breeding status and geographic regions-based classification, respectively.. Among populations, the fixation index of 0.83 and 0.84 was observed due to geographic regions and breeding status, respectively.

**Table 5:** Analysis of molecular variance for common bean genotypes based on geographic origin and breeding status

Source of variation	df	Sum of squares	Variance component	Percentage of variation	Fixation indices	*P*-value
Based on geographical regions						
Among populations	5	258,892.0	538.8 Va	23.53	FIS = 0.83	FIS = 0.001
Within populations	281	901,478.3	1457.5 Vb	63.67	FST = 0.24	FST = 0.001
Within individuals	287	84,100.5	293.0 Vc	12.80	FIT = 0.87	FIT = 0.001
Total	573	1,244,470.8	2289.4			
Based on breeding status						
Among populations	1	198,461	680.13 Va	27.06	FIS = 0.84	FIS = 0.001
Within populations	287	968,117	1540.49 Vb	61.3	FST = 0.27	FST = 0.001
Within individuals	289	84,463.5	292.26 Vc	11.63	FIT = 0.88	FIT = 0.001
Total	577	1,251,041	2512.88			

*Df*: Degree of freedom

### Genetic diversity and population structure

**Fig. 5:**
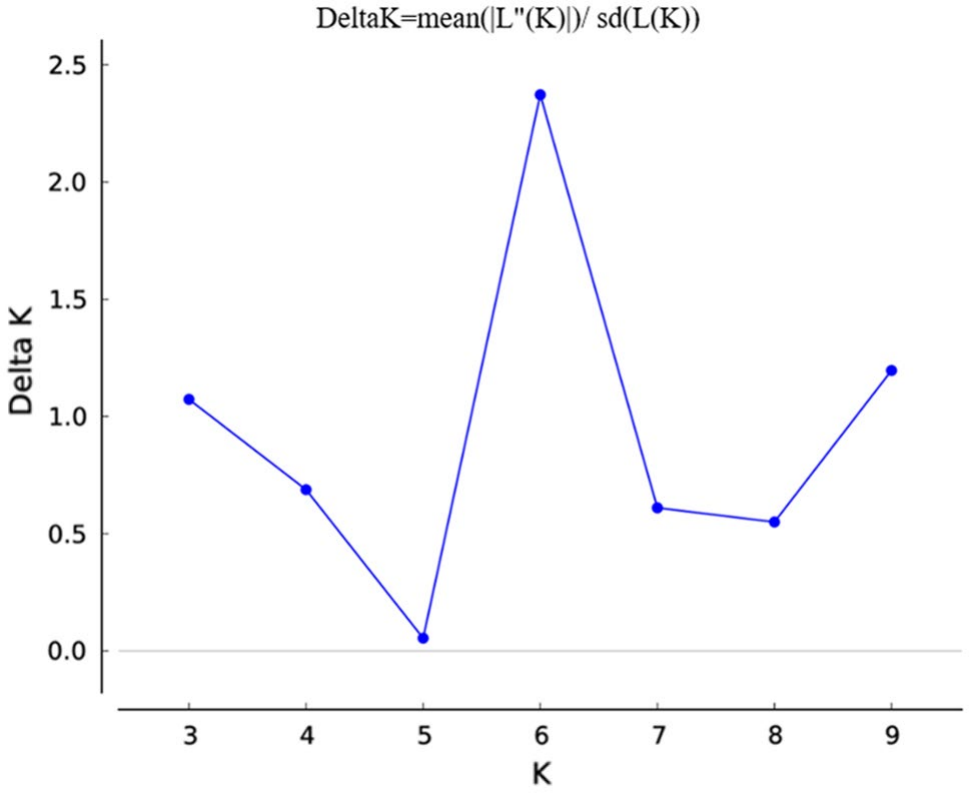
Optimal *K* value to group the 289 common bean genotypes into subpopulations.

**Fig. 6:**
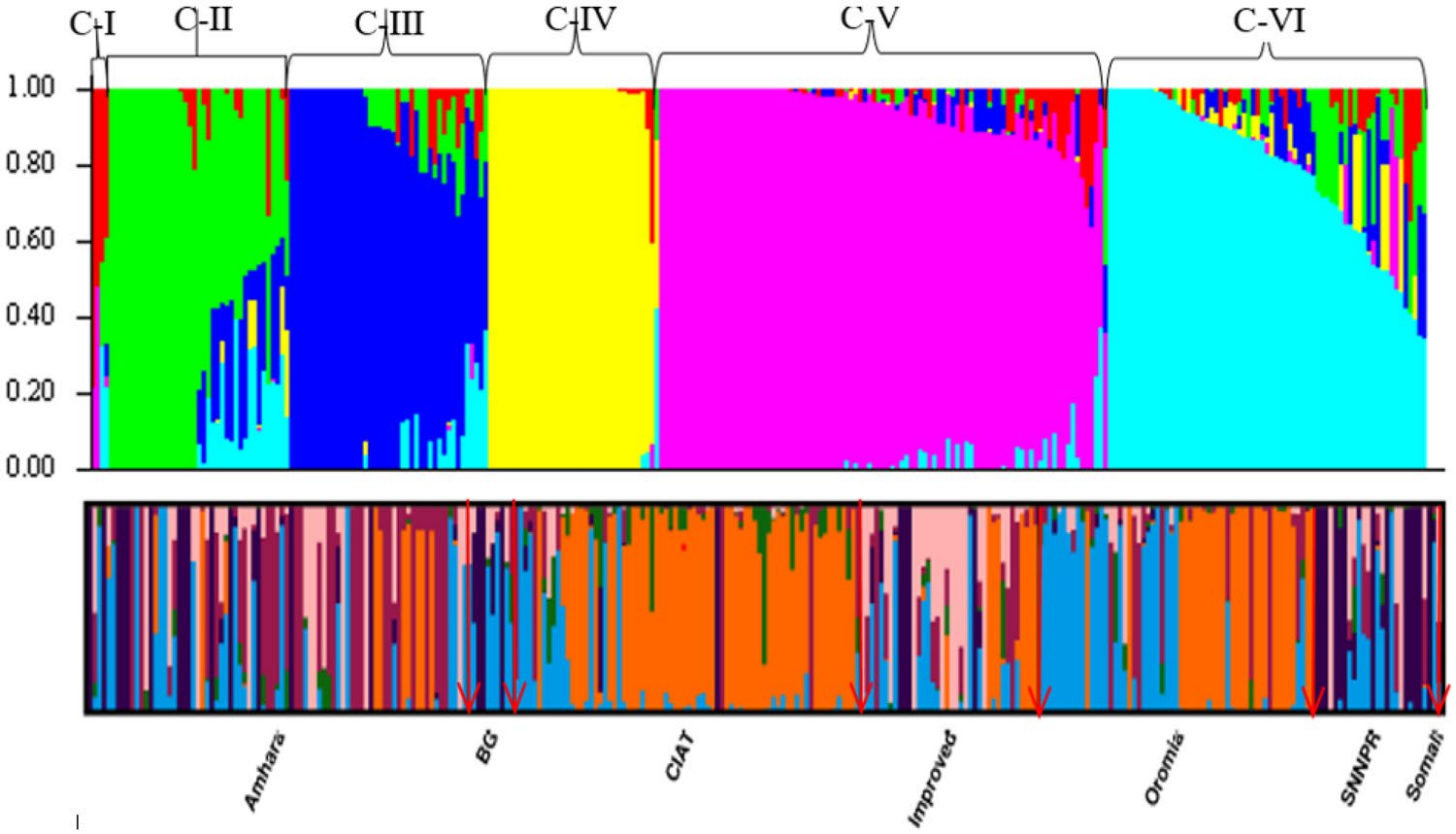
Population structure for 289 common bean genotypes in *K* = 6. Each color represented a cluster. The arrow indicates the delineation of the genotypes based on regions of collection at *K* = 6. *BG*: Benshangul Gumuz, *CIAT*: International Centre for Tropical Agriculture, *SNNPR*: Southern Nations Nationalities and People Region. The arrows demarket the genotypes in each region.

The optimal *K* value to group the common bean genotypes into subpopulations was found at *K* = 6 (Fig. 5). In the first level of subpopulation grouping, *K* = 2, the common bean genotypes were roughly classified into Andean and Mesoamerican genepools (Fig. 5). One hundred and eighty-eight (65.05%) genotypes were assigned in the Mesoamerican group, while the remaining 96 (33.22%) genotypes were classified in the Andean genepool. (Fig. 6).

The remaining five genotypes were admixed including three landraces, namely SWM012, 211,266, 211,305, and two breeding lines from CIAT, *i.e*., NUS-1 and NUA-355. The threshold of membership coefficient (qi) to assign genotypes with a group was considered as qi ≥ 0.70. The known high seed Fe concentration line MIB 465 was assigned in the Mesoamerican genepool, while RWR2154 and CAL-96 were included in the Andean genepool as reference lines in the respective genepool.

At *K* = 6, the Mesoamerican genepool was separated into five distinct classes, each subpopulation consisting of 35, 21, 35, 1, and 50 genotypes (coded as MA1, MA2, MA3, MA4, and MA5, respectively (Fig. 6). Fifty-five genotypes were considered as admixture. The Andean genepool (A1) remains unseparated, except four genotypes. The first subpopulation (MA1) of the Mesoamerican genepool consisted of genotypes mainly collected from Amhara region and were small seeded. The second subpopulation (MA2) consisted of landraces mainly collected from Amhara and Oromia region. The third subpopulation (MA3) consisted of landraces mainly collected from Amhara and SNNPR. The fourth subpopulation (MA4) consisted of a single genotype, NUA-355 from CIAT. The fifth subpopulation (MA5) consisted of small- and medium-seeded improved varieties and breeding lines and landraces mainly from Amhara regions and few from Benshangul Gumuz and SNNPR regions.

To further confirm the genetic structure of the genotypes, principal component analysis was employed and the first two principal components explained 91% of the total variation (Figure S1). The genotypes were grouped into Andean and Mesoamerican genepools with some admixture.

The principal coordinate analysis (PCoA) revealed that the genotypes were clustered based on the population structure result but the genotypes were not distinct due to the presence of admixture and gene flow among the different subpopulations particularly in the Mesoamerican genepool (Figure S2).

The net nucleotide distance among the six subpopulations generated based on population structure revealed that the maximum distance was recorded between population five and six, while the lowest distance was found between population three with population four and five (Table 6). The highest expected heterozygosity (0.28) was found in population two, while the lowest (0.01) was in population five. Similarly, mean fixation index among the populations ranged from 0.60 (Pop2) to 0.95 (Pop 5).

**Table 6:** Net nucleotide distance, expected heterozygosity and mean fixation index among the common bean populations

Net nucleotide distance							Expected heterozygosity	% of membership
Population	Pop1	Pop2	Pop3	Pop4	Pop5	Pop6		
Pop1							0.03	0.14
Pop2	0.37						0.28	0.19
Pop3	0.13	0.33					0.12	0.2
Pop4	0.14	0.34	0.1				0.12	0.28
Pop5	0.17	0.39	0.1	0.15			0.01	0.17
Pop6	0.48	0.48	0.4	0.43	0.5		0.08	0.03

*Pop*: population and each population numbering (1–5) showed MA1 to MA5, respectively, and Pop6 is A1

## Discussion

### SNP distribution and variation

Information on genetic diversity and relationship among common bean genotypes helps in the sustainable and efficient utilization of common bean genetic resources by breeding programs. Assessing the genetic diversity is very vital to get a better understanding of sources of novel alleles to be used in the crop improvement program. Various types of molecular markers have been widely used to estimate genetic diversity and population structure of common bean [43, 44]. Currently, DArTSeq-based SNP markers became a subject of choice because of high-throughput, high-genome coverage and are less costly [45].

Previously, studies were carried out to characterize the common bean genotypes in different countries and sources of germplasm from wild to domesticated using different molecular markers [46, 47]. However, few studies are available on the use of high-density DArTSeq SNP markers for diversity and population structure study in common bean [22, 48].

The distribution of the DArTSeq markers was almost uniform across the chromosomes with the largest number of SNPs recorded in chr_2 (1280 SNPs), followed by chr_8 (1272 SNPs), while the minimum SNPs were recorded in chr_4 (835). Consistent to this study, [48-50] reported higher number of SNPs on chr_2.

Transition to transversion (Ts/Tv) ratio is one of the methods to check the quality of the SNP calls. The studied 289 common bean genome revealed that A/G and T/C were the most prevalent with a Ts/Tv ratio of 1.39:1 suggesting the transition mutations were more common than transversion mutation following methylation. The result is in agreement with [50] who reported a Ts/Tv ratio of 1.24 in 188 Brazilian common bean germplasm using 6286 SNP markers. Similarly, [22] reported a Ts/Tv ratio of 1.75 in 50 Italian common bean germplasm using 11,866 SNP markers.

In the present study, the overall mean genetic diversity, heterozygosity, MAF, and inbreeding coefficient were 0.38, 0.05, 0.28, and 0.85, respectively. This showed that the studied common bean genotypes have a wide diversity that can be exploited in the common bean improvement program. Contrary to this study [51] reported lower values for heterozygosity and genetic diversity after a study of 183 Turkish common bean accessions. In agreement with this study, [52] studied 60 common bean genotypes from eastern and southern Africa using 16,565 silicoDArT markers and reported overall genetic distance, observed heterozygosity and MAF of 0.28, 0.45, and 0.24, respectively.

### Genetic diversity and relationship

The highest heterozygosity (6.0%) was observed in the improved varieties compared with landraces collected from different regions of Ethiopia. The result was further confirmed by the highest heterozygosity of materials sourced from CIAT and improved varieties with respective heterozygosity of 8 and 4.69% in the geographic origin-based classification. This indicated that the landraces were almost fixed to homozygosity compared with the improved varieties.

Polymorphic information content that measures the relevance of markers in a diversity study ranged from 0.06 to 0.38 with an average of 0.30. The maximum PIC in SNP markers is 0.50 due to its bi-allelic nature. Accordingly, the SNP markers used in this study were highly informative compared with other studies [49] and [48] who reported respective PIC values of 0.25 and 0.22 in common bean. The higher PIC value in this study could be due to the higher number of genotypes obtained from diverse sources and large number of SNP markers. For multi-allelic markers (e.g., microsatellites), PIC is regarded as slightly informative, moderately informative, and highly informative when the PIC value is < 0.25, 0.25–0.50 and > 0.50, respectively [53]. The relatively lower PIC value of SNP markers could be compensated with their large number and wide-genome coverage.

Based on the breeding status, the highest genetic diversity and PIC were observed in the improved varieties compared with the landraces. From the landraces, those collected from Oromia and Amhara region showed higher genetic diversity and PIC. The higher genetic diversity of landraces collected from Oromia region indicated that landraces could have useful traits which pave the way for the collection of additional landraces in areas where there is an expected rich genetic diversity.

Pre-grouping of the genotypes showed that the highest genetic distance was observed between genotypes collected from CIAT and SNNPR (0.49). The CIAT genotypes were closer to improved varieties than all other regions of collection. This supports the fact that many of the improved varieties in Ethiopia and Africa are introduced from the CIAT collections and breeding lines and many of them have close pedigree [54, 55]. Clustering of the common bean genotypes revealed that they were not clustered based on their geographical origin and no clear explanation was given for the grouping and the result is in agreement with [6].

The AMOVA was conducted to display the variation among and within the *K* = 6 subgroups and the result indicated that the greatest variability was observed within populations in both geographical region and breeding status-based classification followed by among populations.

### Population structure

In the present study, the highest delta *K* value occurred at *K* = 6 and grouped the 289 common bean genotypes into six subpopulations with a membership coefficient (qi) ≥ 0.70. At *K* = 2, the genotypes were classified into Andean and Mesoamerican genepool. The classification was mostly based on seed size in which Mesoamerican genotypes contained genotypes with small to medium seed size, while the Andean genotypes contained genotypes with medium to large seed size. At *K* = 6, the Mesoamerican genepool further separated into five subclusters, while the Andean genepool remains the same as the *K* value was increasing, except four genotypes. A second peak was observed at *K* = 9 which might be associated with the presence of different races within the Andean and Mesoamerican genepool. In agreement with this study, two peaks were reported in common bean at *K* = 3 and *K* = 7 [18, 28, 42, 43] have also reported two peaks. In addition, the membership coefficient revealed that there were admixtures that could be associated with the background of the genotypes used in this study as common bean genotypes might be hybridized for different traits.

In Ethiopia, the majority of the landraces and commercial cultivars were Mesoamerican type and are widely cultivated both for consumption and export, while the Andean genepool (large seeded) have relatively shorter period of cultivation history and the majority of them were introduced from CIAT as breeding lines and some are released after multi-location trial. In addition, the longtime cultivation and planting of mixed landraces in the same plot may result in the variation among the Mesoamerican genepool. The population structure detected by delta *K* varied with the number of populations, source of germplasm, and number and type of markers used. Accordingly, various studies reported different delta K values. In other studies, an optimal population structure at *K* = 6 was reported [6, 56].

The eastern African region is considered as the secondary center of origin of common bean which could be the reason for such wide population structure [6]. In the present study, at *K* = 6, the Mesoamerican genepool was separated into five subpopulations, while the Andean genepool remained unseparated. In agreement with this result, [44] reported the Mesoamerican genepool was separated into four subpopulations at *K* = 5, while the Andeans remained unseparated. The results of population structure revealed that the Mesoamerican genepool was more diverse than the Andean genepool. This could be due to large sample size of Mesoamerican genepool and long years of cultivation in Ethiopia under different cropping system.

## Conclusions

The present study revealed the presence of wide genetic diversity and structured population in common bean. The average overall genetic diversity, PIC, MAF, and inbreeding coefficient were 0.38, 0.30, 0.28, and 0.85, respectively. Genetic diversity based on geographical locations showed that landraces collected from Oromia region showed the highest genetic diversity, PIC, and inbreeding coefficient.

Pre-grouping of the genotypes based on geographical origin showed that genotypes form SNNPR and CIAT were the most distant (0.492). Genotypes collected from CIAT were distant than genotypes collected from regions in Ethiopia. However, it is relatively close to the improved varieties due to sharing of parents in the improvement process. Clustering also showed that the genotypes were not clustered based on their origin, which could be due to transboundary movement of the germplasm across the regions. This showed that geographical regions were not the main drivers for differentiation and selection of the parents should be based on systematic assessment of the diversity. The population structure revealed that the optimal number of subpopulation was six (*K* = 6). The results showed that common bean collections revealed a wide range of diversity. At *K* = 2, the genotypes were clustered into Andean and Mesoamerican genepool. Further separation of the genotypes was due to the diversity, large sample size, and long years of cultivation of the Mesoamerican genepool, while the Andeans remained unseparated. The genotypes were not clustered based on geographical regions and they were not the main drivers for the differentiation and. This indicated that selection of the parental lines should be based on systematic assessment of the diversity rather than geographical distance.

The present investigation proved the potential of highly informative DArTSeq-derived SNP markers for population structure and genetic diversity studies. From this study, a core set of diagnostic SNP markers can be built and used in the genetic diversity and population structure of common bean for association studies, designing effective collection and conservation for efficient utilization for the improvement of the crop.

## Supplementary Information

Below is the link to the electronic supplementary material.

Supplementary file1 (DOCX 15 KB)

Supplementary file2 (TXT 10418 KB)

Supplementary file3 (DOCX 105 KB)

Supplementary file4 (DOCX 334 KB)
