# Supplementary material for: High-density DArTSeq SNP markers revealed wide genetic diversity and structured population in common bean (Phaseolus vulgaris L.) germplasm in Ethiopia
Source: Mol Biol Rep. 2023 Jun 30;50(8):6739–51. doi: 10.1007/s11033-023-08498-y (PMC10374692; doi:10.1007/s11033-023-08498-y)
Supplement: Supplementary file 1 — Supplementary file1 (DOCX 15 KB) [file 11033_2023_8498_MOESM1_ESM.docx]

Table S1. Collection region, number of genotypes and altitudinal range of the collection sites

| Geographic region | Zone of collection | Districts of collection | List of genotypes | No, of genotypes | Altitudinal range |
| --- | --- | --- | --- | --- | --- |
| Amhara | East Gojjam, Oromia zone, South Wello, West Gojjam | Enbise Sar Midir, Shebel Berenta, Huleteju nese, Goncha siso nese, Bati, Simada, Gaynet, Kombolcha, Mekdela, Tehuledere, Kalu, Debresina, Sayinit, Wogidi, Bure Wemberma, Jabi Tehnan, Yelmana Densa, Bahrdar Zuria, | 228522, EGA 034, EGA 035, EGA 036, EGAA038, EGAA039, EGB031, EGB032, EGB033, EGL040, EGL041, EGL042, EGY037, EGZ043, EGZ044, EGZ045, EGZ046, NC-02, NC-03, NC-05, NC-07, NC-10, NC-16, NC-17, 215719, 215720, 207534, NC-25, NC-28, NC-29, NC-30, NC-34, NC-31, NC-39, NC-44, NC-48, NC-49, NC-50, NC-51, NC-52, NC-53, NC-54, NC-57, NC-61, SWD016, SWD017, SWD018, SWD019, SWD020, SWD021, SWDO022, SWDO023, SWM012, SWM013, SWM014, SWM015, SWM024, SWM025, SWM026, SWM027, SWM028, SWM029, SWM030, SWS001, SWS002, SWS003, SWS004, SWS005, SWZ 006, SWZ 007, SWZ 008, SWZ 009, SWZ 010, SWZ 011, 211266, 211267, 211269, NC-08, NC-09, NC-12, NC-13, NC-14, NC-15, NC-18, NC-20 | 85 | 1838-2447 |
| Benshangul Gumuz | Assosa, Metekel | Assosa,  Dangur,  Dibate | 207934, 207938, 207935, 211346, 211347, 211348, 211349, 211356, 211361, 211362 | 10 | 1000-1400 |
| Oromia | Bale, Borena, East Hararghe, Illubabor, Jimma, North Omo, West Hararghe, West Wellega | Ginir, Arsi, Yabelo, Teltele, Meta, Goro Gutu, Deder, Bure, Darimu, Kersa, Mana, Illubabor, Tulo, Chiro, Guba Koricha, Doba, Sayo, Gawo Dale | 215391, 230044, 230526, 230661, 232196, 237079, 237080, 215048, 215049, 228812, 211315, 211320, 211323, 211333, 211314, 219231, 228911, 228913, 213197, 201066, 241134, 212978, 211302, 211311, 211325, 211305, 211306, 208638, 208699, 211304, 223329, 228813, 208702, 208703, 208705, 208995, 211340, Small white | 38 | 1846-2345 |
| SNNPR | Benchi Maji, Hadiya, Kembata Tembaro, North Omo, Sidama, South Omo | Konso special, Badawacho, Omo sheleko, Kacha bira, Alaba, Damot Gale, Boreda Abaya, Kindo Koysha, Boloso Sore, Bako Gazer | 214663, 214664, 214665, 214675, 214676, 214678, 215051, 213046, 241752, 241757, 241756, 241736, 228077, 228082, 228086, 228085, 241748, 244805, 211546, 211552, 237993, 241739, 241734, 208367, 211279, 211280, 211284, 211286, 212860 | 29 | 800-2880 |
| Somali | Jijiga, Shinile | Jigjiga | 230525, 211331 | 2 | 1850-1930 |
| Improved varieties |  |  | SAB 632, Anger, Argene, Atndaba, Awash 1, Awash Melka, Awash Mitin, Awash-2, Ayenew, Babile, Batu, Beshbesh, Bifort large seeded-5, Brazil-2, Chercher, Chore, Cranscope, DAB-107, Deme, Dimtu, Dinknesh, Dursitu, F10 sel new bilfa 58, Fedis, Gabisa, GLP-2, Gobe Rasha-1, Gofta, Haramaya, Hawassa Dume, Hirna, Hundane, Ibado, KAT-B1, KAT-B9, Kufanziq, Lehode, Loko, Melka Dima, Mexican-142, Montcalm, Morka, Nasir, Nazareth-2, Omo-95, Ramada, Red Wolaita, Roba-1, SAB 736, SARI-1, SER-119, SER-125, Tabor, Tatu, Tibe, Tininke, USDK, Waju, Wedo | 59 |  |
| CIAT lines |  |  | CAL96, CBB1, DAN-10, DOR 500, KAB06F2.8-27, MIB 465, NUA 714, NUA 739, NUA 94, NUA 225, NUA 345, NUA 347, NUA 355, NUA 497, NUA 56, NUA 561, NUA 577, NUA 582, NUA 59, NUA 605, NUA 615, NUA 636, NUA 648, NUA 69, NUA 718, NUA 742, NUA 81, NUA-186, NUA 227, NUA 229, NUA 230, NUA 350, NUA 511, NUA 512, NUA 513, NUA 514, NUA 515, NUA 517, NUA 527, NUA 528, NUA 541, NUA 560, NUA 561, NUA 576, NUA 99, NUS-1, NUS-12, NUS-17, NUS-18, NUS-25, NUS-27, NUS-30, NUS-33, NUS-6, NUS-8, NUS-9, RWR2154, Vax 1, Vax 2, Vax 3, Vax4, DAB-410, DAB-370, Candidate 1, SCN-11, SCN-5, SCR-11, SCR-15, Candidate 2, RAZ-11, RAZ-42, Candidate 3, candidate 4, candidate 5 | 74 |  |
