# Supplementary material for: High-density DArTSeq SNP markers revealed wide genetic diversity and structured population in common bean (Phaseolus vulgaris L.) germplasm in Ethiopia
Source: Mol Biol Rep. 2023 Jun 30;50(8):6739–51. doi: 10.1007/s11033-023-08498-y (PMC10374692; doi:10.1007/s11033-023-08498-y)
Supplement: Supplementary file 3 — Supplementary file3 (DOCX 105 KB) [file 11033_2023_8498_MOESM3_ESM.docx]

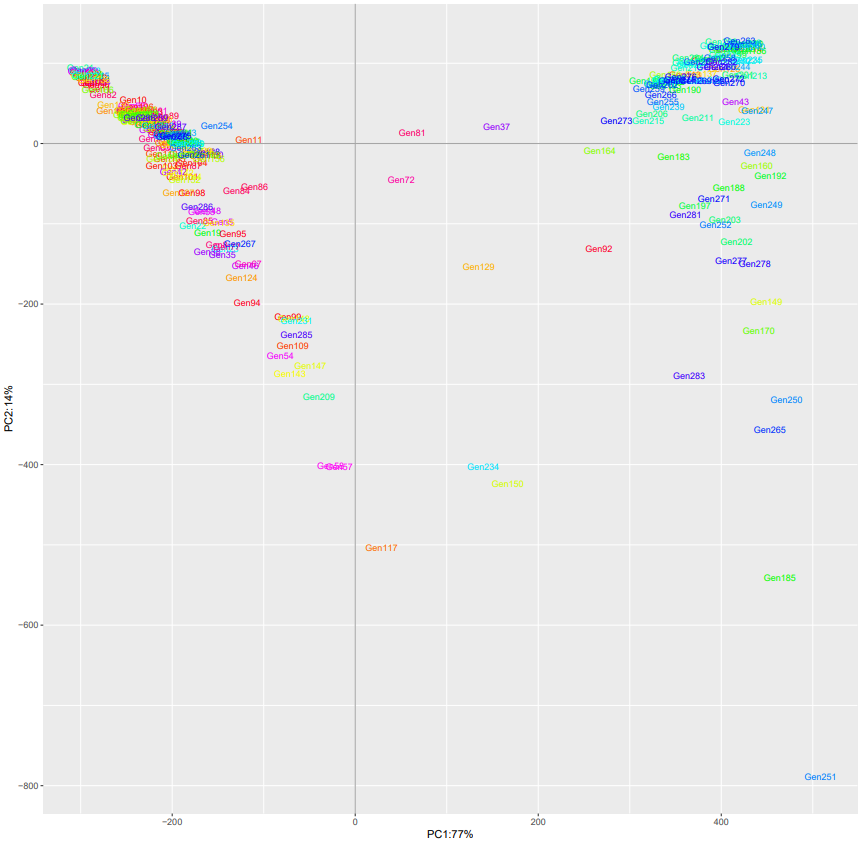


**Figure S1. Principal component analysis of the populations of 289 common bean genotypes based on 11480 SNPs. Each genotype is represented by sample number and each sample color corresponding to the assigned subgroup.**
