# Supplementary material for: High-density DArTSeq SNP markers revealed wide genetic diversity and structured population in common bean (Phaseolus vulgaris L.) germplasm in Ethiopia
Source: Mol Biol Rep. 2023 Jun 30;50(8):6739–51. doi: 10.1007/s11033-023-08498-y (PMC10374692; doi:10.1007/s11033-023-08498-y)
Supplement: Supplementary file 4 — Supplementary file4 (DOCX 334 KB) [file 11033_2023_8498_MOESM4_ESM.docx]

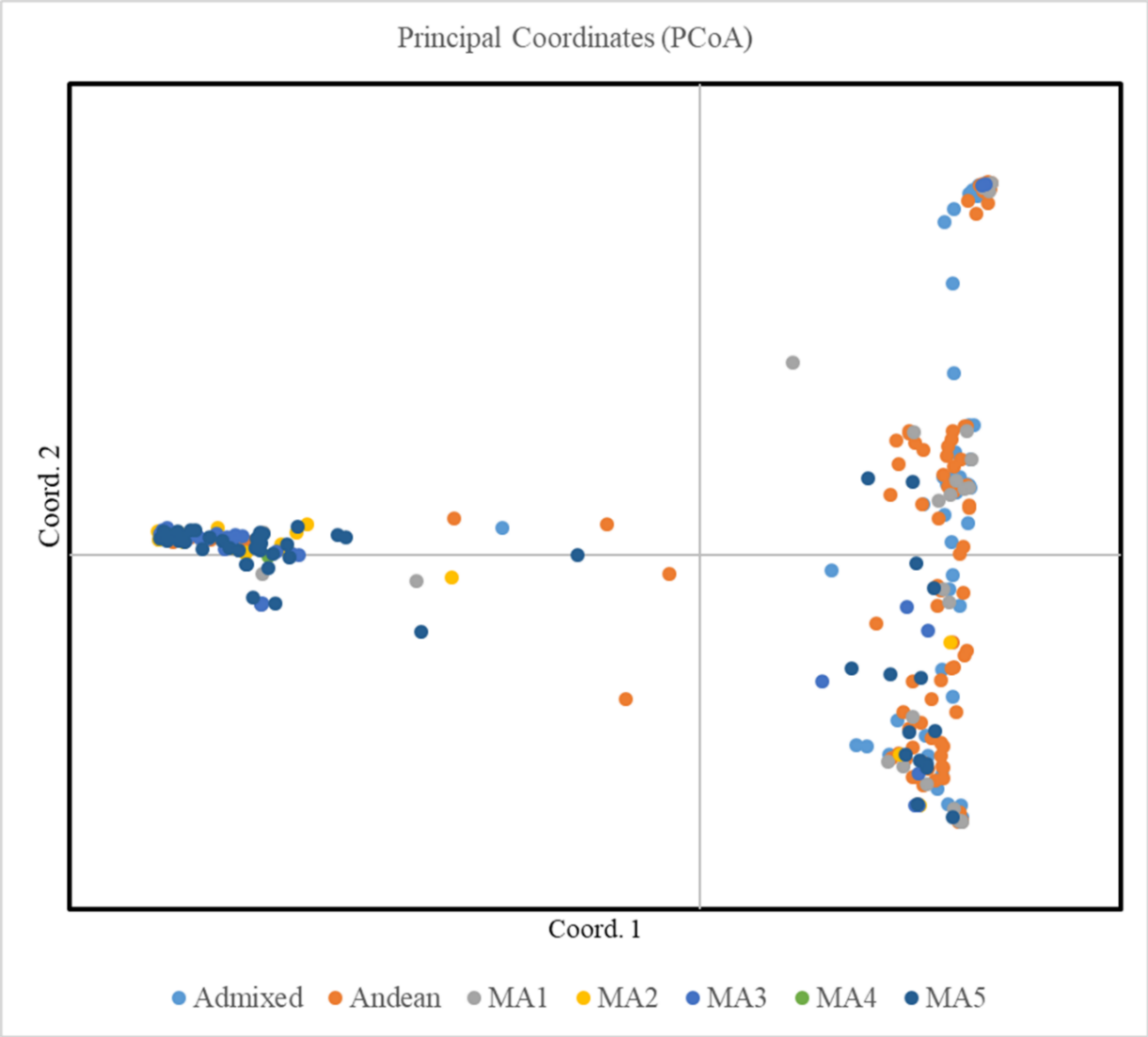


Figure S2. Principal coordinate analysis of the common bean subpopulations showing the distribution of the populations along the axis.
